# Doxorubicin Influences the Expression of Glucosylceramide Synthase in Invasive Ductal Breast Cancer

**DOI:** 10.1371/journal.pone.0048492

**Published:** 2012-11-02

**Authors:** Xiaofang Zhang, Xiaojuan Wu, Peng Su, Yongsheng Gao, Bin Meng, Yanlin Sun, Li Li, Zhiqiang Zhou, Gengyin Zhou

**Affiliations:** 1 Department of Pathology, Shandong University School of Medicine, Jinan, Shandong, P.R. China; 2 Department of Pathology, Academy of Tumor Treatment and Prevention, Jinan, Shandong, P.R.China; Faculty of Pharmacy, Ain Shams University, Egypt

## Abstract

**Introduction:**

Glucosylceramide synthase (GCS) is one enzyme that provides a major route for ceramide clearance. Recent evidence has indicated an important role for GCS in multidrug resistance (MDR) tumors. Doxorubicin (DOX)can modulate the expression of GCS in leukemia and ovary cell lines. However, few studies have investigated their relationship in breast cancer;

**Methods:**

We collected 84 excision biopsies from patients with invasive ductal breast cancer of whom 33 patients had undergone preoperative chemotherapy. Immunohistochemistry was used to analyze the expression of GCS protein and significantly showed that the expression of GCS was higher in the samples from patients treated with preoperative chemotherapy(p = 0.018). In order to investigate the underlying mechanism, breast cancer cell lines were cultured with different concentrations of DOX, and mRNA and protein levels of GCS were then detected;

**Results:**

DOX significantly upregulated the expression of GCS at both the mRNA and protein level in ERα-positive MCF-7 cells.We then block down the Sp1 site of GCS promoter, which inhibited the DOX-mediated increase in GCS expression; and after Erα was inhibited in MCF-7 cells, the up-regulation of GCS by DOX also been inhibited.

**Conclusions:**

In conclusion, our data demonstrated the novel finding that DOX could modulate the expression of GCS through the Sp1 site of GCS promoter in ERα-positive breast cancer cells.

## Introduction

Breast cancer is one of the most common causes of death in women due to cancer worldwide. Besides surgical methods, chemotherapy and endocrine therapies are also used in the treatment of breast cancer. The resistance of tumors to chemotherapy occurs not only to single cytotoxic drugs, but also as a cross-resistance to a range of drugs with different structures and cellular targets. This phenomenon is termed multidrug resistance (MDR), and is one of the contributing factors that prevents survival rates for breast cancer improving further [1]. Several factors have been reported to be responsible for MDR, including the overexpression of the adenosine triphosphate (ATP)-binding cassette (ABC) membrane transporter family [2].

The accumulation of recent evidence has pointed towards an important role for glucosylceramide synthase (GCS) in MDR. Sphingolipids, which include ceramide and sphingosine, are essential structural components of cell membranes. Furthermore, they also play an important role in regulating the proliferation, survival and apoptosis of cells. GCS is a pivotal enzyme that transfers UDP–glucose to ceramide to form glucosylceramide (GC) [3]. We and others have shown that MDR cancer cells have high levels of GCS compared to drug-sensitive cells [4], [5]. Transfection with GCS could increase the level of MDR in breast cancer cell lines [6], whereas its inhibition has proven to be useful in altering responses to chemotherapy in numerous human tumor cell lines [5], [7].

Anthracycline-based chemotherapy (treatment regimens that involve anthracyclines such as doxorubicin or epirubicin) has been used clinically for over two decades. Several studies have confirmed that doxorubicin (adriamycin) can modulate the expression of GCS in the leukemia cell line, HL-60, and an ovary cell line, NCI/ADR-RES [8], [9]. Few studies have shown whether doxorubicin influences the expression of GCS in breast cancer tissue samples and breast cancer cells. This study aimed to rectify this omission from the literature.

## Materials and Methods

### Clinical Samples

Tissue samples from 84 patients with invasive ductal breast carcinoma who underwent complete dissection of the breast and axillary lymph nodes and 5 patients with accessory breast who underwent complete dissection of the tissue were collected at the Qilu Hospital Shandong University, China, between January and September 2007. Thirty-three of the patients had also undergone preoperative chemotherapy (CAF protocol: cyclophosphamide, doxorubicin and 5-fluorouracil).

Tumor samples were paraffin-embedded and histopathological variables, including tumor size, lymph node metastasis, histological subtype, and histological grade were determined by reviewing pathology reports and hematoxylin and eosin (H&E) stained sections. Patient and tumor characteristics are summarized in Table 1.

**Table 1:** Patients and tumor characteristics for the 196 reference invasive ductal breast cancer data series.

Characteristics	Number of patients (%)
**Age**	
≤35	6 (7.10)
35–60	64(76.2)
>60	14 (16.7)
**Tumor stage**	
T1–2	77(91.7)
T3–4	7 (8.30)
**Nodal stage**	
N0	44(52.4)
N1−x	40(47.6)
**Histologic grade**	
Grade I	8(9.50)
Grade II	62(73.8)
GradeIII	14(16.7)
**ERα**	
Negative	29(34.5)
Positive	55(65.5)
**Pre-operation chemotherapy**	
Yes	33(39.3)
No	51(60.7)

ERα -estrogen receptor α.

The use of these tissues was approved by the Research Ethics Committee of Shandong Medical University, and we obtained informed written consent for pathological evaluation from all participants involved in our study.

### Cell Culture

Two drug-sensitive breast cancer cell lines, MCF-7 (ER-positive) and MDA-MB-231 (ER-negative), were obtained from the American National Cancer Institute. The multidrug-resistance breast cancer cell line, MCF-7/ADM, was selected from MCF-7 using doxorubicin treatment in stages [10]. All the cells were maintained in RPMI-1640 medium (Gibco, USA) containing 10% FBS at 37°C in a humidified atmosphere containing 5% CO_2_.

**Figure 1:**
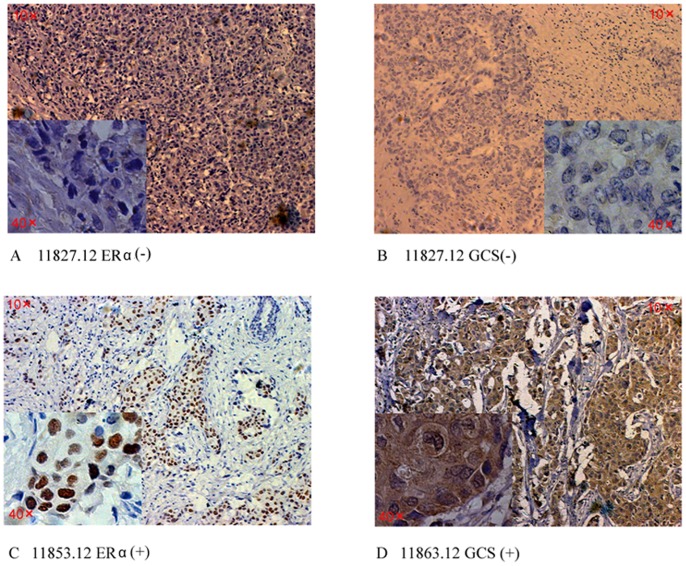
Expression of GCS and ERα protein in invasive ductal breast carcinoma samples. The expression of GCS and ERα protein was detected in all samples by immunohistochemical staining. For GCS, cytoplasmic staining was considered positive; and for ERα, nuclei staining was considered positive. The figure shows two cases that were positive or negative respectively. A, B) An invasive ductal breast carcinoma sample that the expression of ERα and GCS were both negative. C,D) An invasive ductal breast carcinoma sample that the expression of ERα and GCS were both positive.

### Immunocytochemical or Immunohistochemical Analyses

Immunohistochemical staining was carried out using the DAKO Envision Detection Kit (Dako, Carpinteria, CA, USA). In brief, tissue blocks were cut into 4 µm-thick sections, dried, deparaffinized, and rehydrated. Antigen retrieval was performed in a microwave oven for 15 min in 10 mM citrate buffer at pH 6.0. For the cell culture experiments, after cells were embedded in 4% neutral formaldehyde for 2 h, PBS with 0.5% Tween-20 was added for 30 min at room temperature. For all samples, endogenous peroxidase activity was blocked with a 3% H_2_O_2_-methanol solution. The slides were blocked with 10% normal goat serum for 10 min and were incubated with GCS antibody (1∶500) overnight at 4°C. The slides were then probed with HRP-labeled polymer conjugated to a secondary antibody for 30 minutes. The antibody against GCS was a kind gift from Dr. D. Marks (Mayo Clinical Center, Rochester, USA). After the MCF-7/ADM cell line was confirmed to overexpress GCS protein, it was used as a positive control in this research. Otherwise, the normal breast tissues were also used as control.

**Figure 2:**
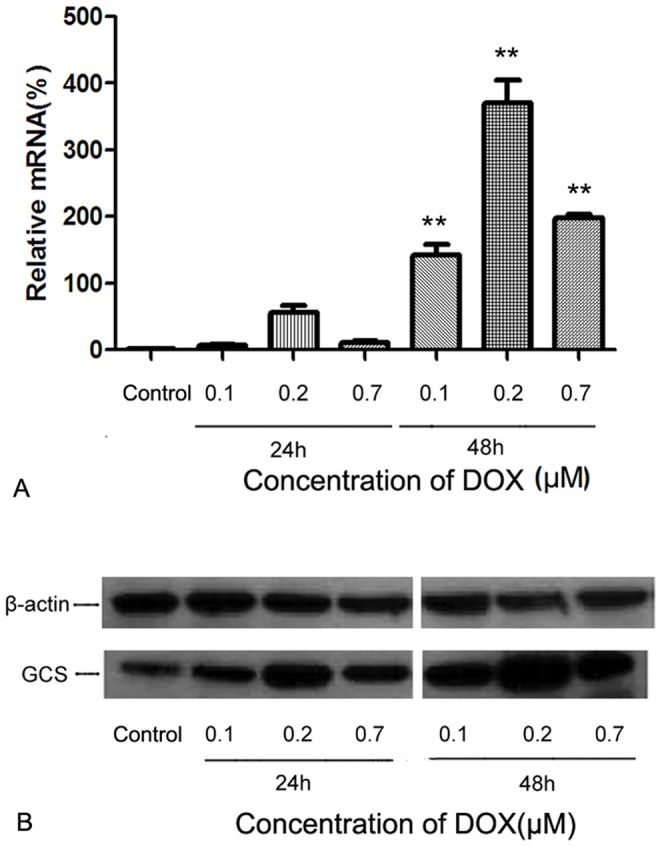
Doxorubicin modulates the expression of GCS mRNA and protein in MCF-7 cells. Cells were seeded at an initial density of 1×10^5^ cells/ml and treated with or without different concentrations of DOX for 24 h or 48 h. Total RNA was extracted and transcribed to mRNA. Then, qPCR was performed to detect the relative mRNA levels of GCS. A) Changes in GCS mRNA levels in MCF-7 cells. B) Changes in GCS mRNA levels in MDA-MB-231 cells. **p<0.01 compared with untreated cells.

Staining results were interpreted by a breast pathologist who was blinded to patient outcomes. Tumors with 1% or more positively stained nuclei were considered positive for estrogen receptor(ER) [11].A dual semi-quantitative scale that combined staining intensity and the percentage of positive cells was used to evaluate GCS protein staining. The staining intensity was scored as 0 (negative), 1 (weak), 2 (moderate), or 3 (strong). The percentage of positive cells was scored as follows: 0, no staining or staining in <5% of the tumor cells; 1, staining in 5% to 25% of the cells; 2, staining in 26% to 50% of the cells; 3, staining in 51% to 75% of the cells; and 4, staining in >75% of the cells. As the overexpression of GCS is related to MDR, cytoplasmic staining was considered positive; an IHC score ≥4 was defined as high expression and <4 was considered to be low expression.

**Figure 3:**
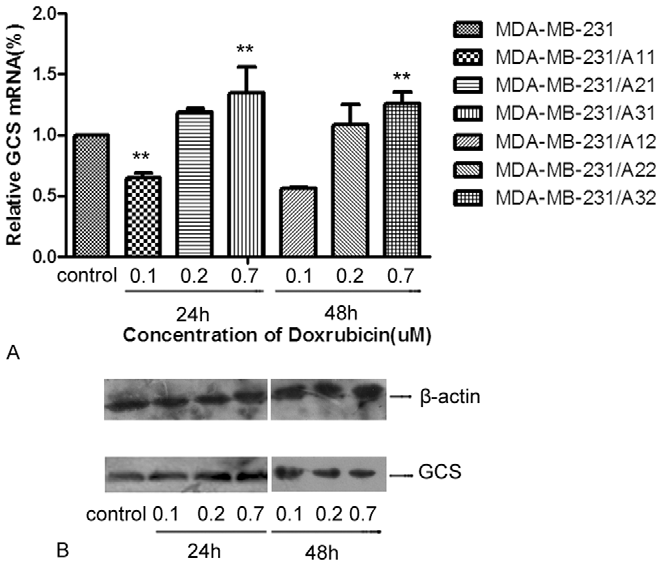
Effects of DOX on GCS mRNA and protein in MDA-MB-231 cells. Cells were seeded at an initial density of 1×10^5^ cells/ml and treated with or without different concentrations of DOX for 24 h or 48 h. Total protein was extracted and Western blotting was used to detect the expression of GCS protein in each group. A) GCS protein levels in MCF-7 cells. After treatment with DOX, GCS levels markedly rose in MCF-7 cells, which was most significant at 0.2 µM DOX for 48 h. B) GCS protein expression in MDA-MB-231 cells. There were no significant changes.

### Treatment with Doxorubicin

Cells were treated with various concentrations of DOX (0.1 µM, 0.2 µM and 0.7 µM) for 24 or 48 hours. The concentrations of DOX were calculated according to the IC50 of the cells (IC50 =  drug concentration (µmol/L) that results in the 50% inhibition of cell growth).

**Figure 4:**
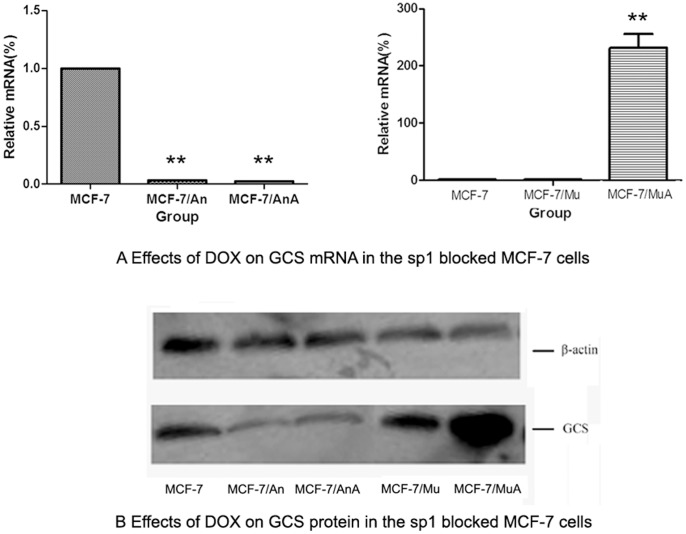
Effects of DOX on GCS mRNA and protein levels in Sp1 blocked MCF-7 cells. Cells were transfected with the Sp1 decoy ODNs or mock ODNs for 24 h. Then, 0.2 µmol/L DOX was added to the cells for 24 h. The GCS mRNA was then analyzed by qPCR. The protein levels of GCS was measured by western blotting. A, B) Effects of DOX on GCS mRNA levels in transfected MCF-7 cells. C) Effects of DOX on GCS protein levels in transfected MCF-7 cells. An, cells transfected with Sp1 ODNs for 24 h; AnA, cells treated with 0.2 µmol/L DOX for 24 h after transfection with Sp1 ODNs; Mu, cells transfected with mock Sp1 ODNs for 24 h; MuA, cells treated with 0.2 µmol/L DOX for 24 h after transfection with mock Sp1 ODNs. **p<0.01 compared with untreated cells.

### Quantitative Real-time PCR (qPCR) to Detect the mRNA of GCS and ERα

Total RNA was isolated using Trizol (Invitrogen, Carlsbad, USA) and quantitative real-time PCR (qPCR) was used to detect GCS mRNA. qPCR was performed using SYBR Green Real-time PCR MasterMix (TOYOBO, Japan). The primers for GCS were as follows: sense: 5′-CCT TTC CTC TCC CCA CCT TCC TCT-3′, antisense: 5′-GGT TTC AGA AGA GAG ACA CCT GGG-3′ [10]; The primers of ERα were as follows: sense: 5′-CCTCCCGCCTTCTACAGGT-3′,antisense: 5′-CACACGGCACAGTAGCGAG-3′ [12]. β-actin (sense: 5′-ACC CCC ACT GAA AAA GAT GA-3′, antisense:5′-ATC TTC AAA CCT CCA TGA TG-3′) was used as an internal control set. The final volume was 20 µl, and an iCycler iQ Real-Time PCR Detection System (Bio-Rad) was used for qPCR. The amplification data were calculated using the ΔΔCq method. The ΔΔCq method was used to calculate relative mRNA expression. The relative target gene expression was calculated using 2-ΔΔCq, where ΔΔCq  =  target Cq - control Cq, ΔΔCq  = ΔCq target -ΔCq calibrator.

**Figure 5:**
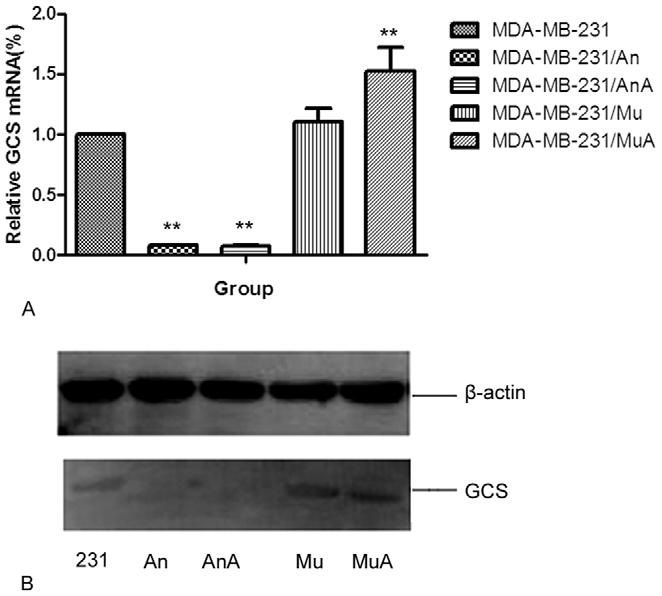
Effects of DOX on GCS mRNA and protein in Sp1 blocked MDA-MB-231 cells. Cells were transfected with the Sp1 decoy ODNs or mock ODNs for 24 h. Then, 0.2 µmol/L DOX was added to the cells for 24 h. The GCS mRNA was then analyzed by qPCR. The protein levels of GCS was measured by western blotting. A) The effects of DOX on GCS mRNA in transfected MDA-MB-231 cells. C) Effects of DOX on GCS protein levels in transfected MDA-MB-231 cells. An, cells transfected with Sp1 ODNs for 24 h; AnA, cells treated with 0.2 µmol/L DOX for 24 h after transfection with Sp1 ODNs; Mu, cells transfected with mock Sp1 ODNs for 24 h; MuA, cells treated with 0.2 µmol/L DOX for 24 h after transfection with mock Sp1 ODNs.

### Western Blot to Analyze GCS Protein Expression

All the cells were cultured for another 48 h after treatment, the confluent cells were then lysed in buffer containing 50 mM Tris-HCl (pH 8.0), 150 mM NaCl, 0.5% Triton-X100, 2 mM EDTA (PH 8.0), 5 mM DTT, 0.2 mM phenylmethylsulfonyl fluoride, and 10 µg/ml aprotinin for 20 min on ice. The complex was centrifuged at 12,000×g for 10 min at 4°C. As described previously [2], equal aliquots of protein (50 µg) were resolved using 4–12% gradient PAGE. The transferred nitrocellulose blot was blocked with 5% fat-free milk powder in TBS at room temperature for 2 h. The membrane was immunoblotted with murine monoclonal antibody C219 against human P-gp (0.7 µg/ml, Santa Cruz) or with GCS-1.2 antiserum (diluted 1∶1,000) in 5% fat-free milk in TBS-0.1% Tween-20. As a control for equivalent protein loading, the filters were simultaneously incubated with rabbit polyclonal antibody against human β-actin (diluted 1∶1,000). Detection was performed using enhanced chemiluminescence (Amersham Pharmacia Biotech, Piscataway, NJ). All analyses were performed in triplicate in three separate experiments.

**Figure 6:**
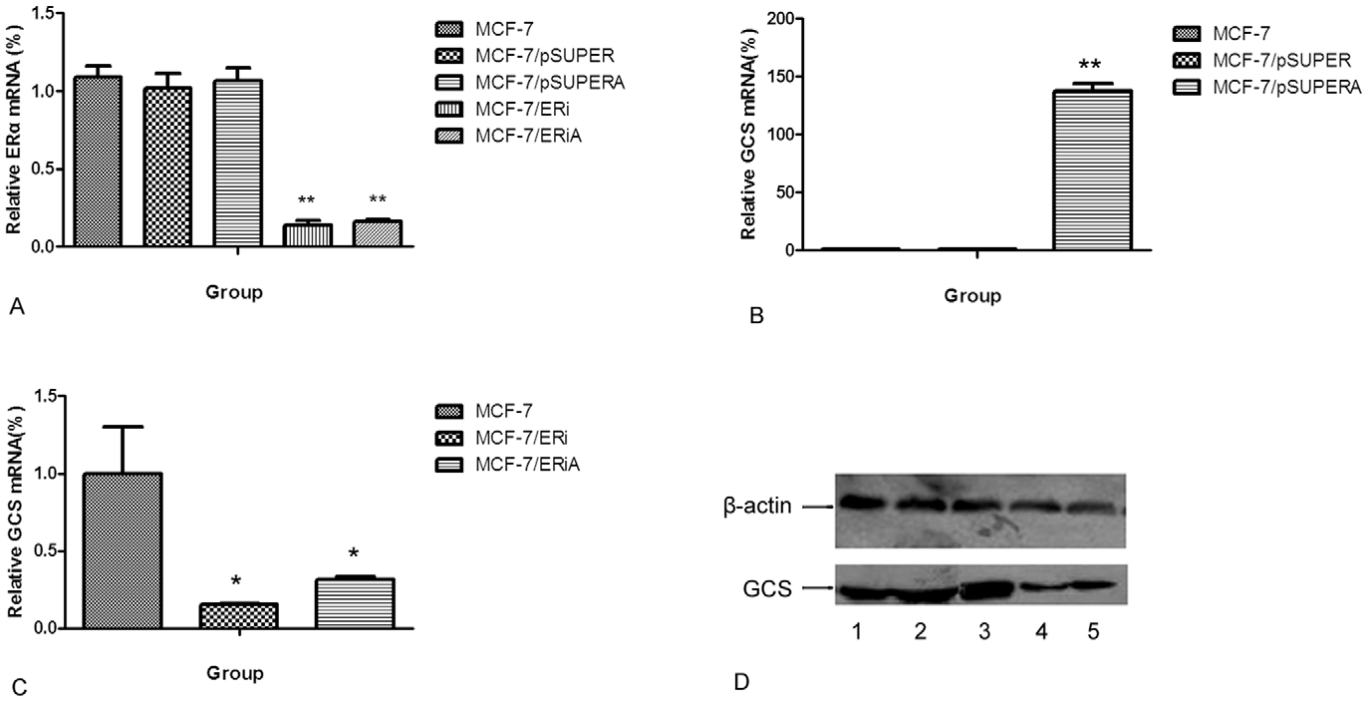
Effects of DOX on GCS mRNA and protein in the ERα blocked MCF-7 cells. Cells were transfected with the plasmid pSUPER-ERαi or pSUPER(control) for 24×h. Then, 0.2 µmol/L DOX was added to the cells for 24 h. The GCS mRNA was then analyzed by qPCR. The protein levels of GCS was measured by western blotting. A) The effects of RNA interference on ERα mRNA in transfected MCF-7 cells. B,C) The effects of DOX on GCS mRNA levels in transfected MCF-7 cells. D) Effects of DOX on GCS protein levels in transfected MCF-7 cells.

### Transfection with GC-rich/Sp1 Decoy Oligodeoxynucleotides

Phosphorothioated double-stranded ODNs (decoy ODNs) containing the sequence of the GCS putative Sp1/GC-rich binding site (5′-ATTCCGGGGGCGGGGGCATG-3′) and mock ODNs (5′-CATGCCATCGCTACCGGGGC-3′) were transfected (final concentration, 0.5 µmol/L) into breast cancer cells suspended in RPMI 1640 using lipofectamine reagent (Invitrogen, Carlsbad, USA) for 12 hours. After transfection, cells were incubated with 10% FCS-RPMI 1640 for 12 hours and then treated with DOX for 24 h [8]. Then, qPCR or Western blotting were used to assess the expression of GCS. Rates of apoptosis were assessed with flow cytometry as described above.

**Figure 7:**
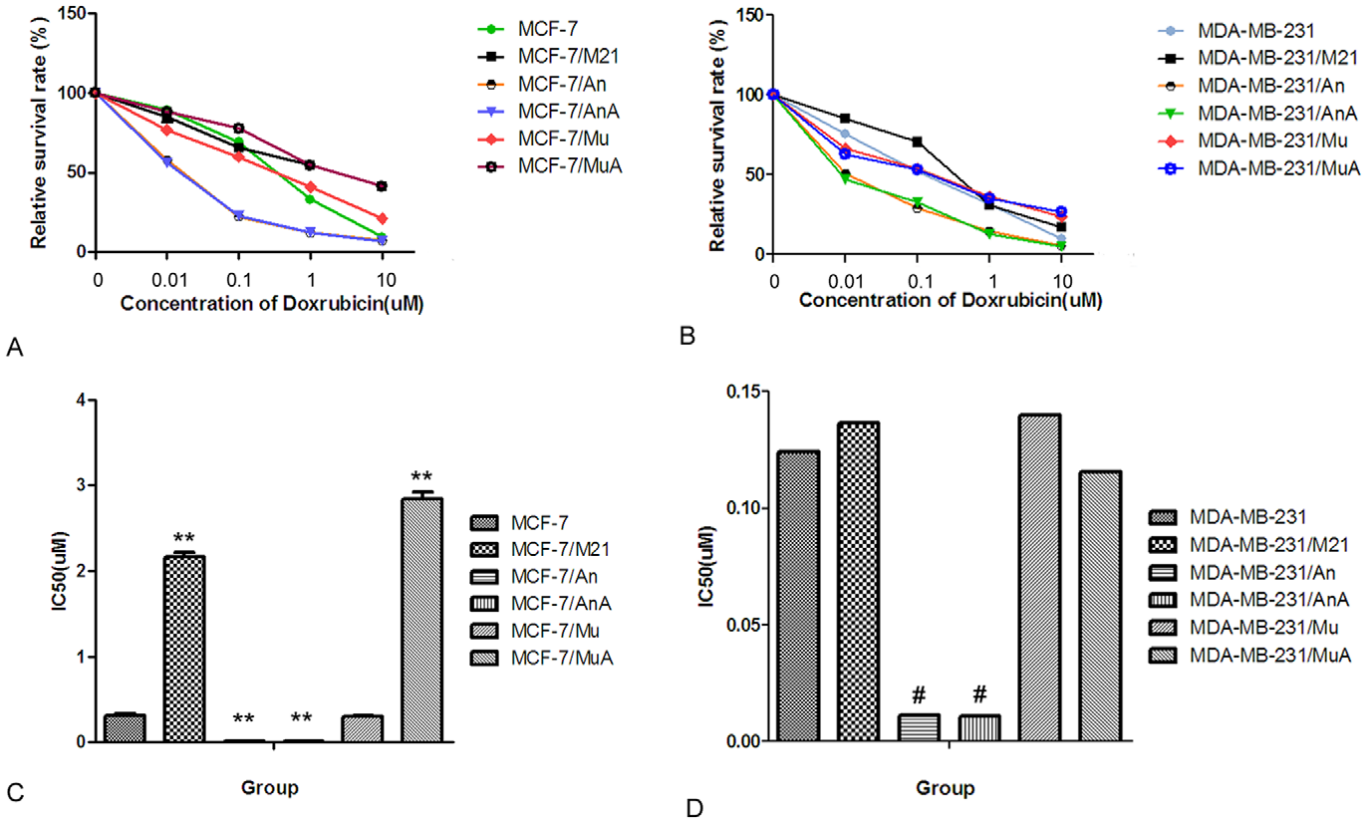
Effects of DOX on IC_50_ values of DOX in each group. Cytotoxicity assays were performed as described in the “Materials and Methods”. The IC_50_ is the drug concentration (µmol/L) that results in a 50% inhibition of cell growth. A, B) The survival curve in MCF-7 cells and MDA-MB-231 cells, respectively.C, D) The IC_50_ value of each group. A21, cells treated with 0.2 µmol/L DOX for 24 h; An, cells transfected with Sp1 ODNs for 24 h; AnA, cells treated with 0.2 µmol/L DOX for 24 h after their transfection with Sp1 ODNs; Mu, cells transfected with mock Sp1 ODNs for 24 h; MuA, cells treated with 0.2 µmol/L DOX for 24 h after they were transfected with mock Sp1 ODNs. **p<0.01 compared with MCF-7 cells. # *p*<0.05 compared with MDA-MB-231 cells.

### Transfection with ERα Interference Plasmid

The RNA interference sequence targeted to ERα was selected according to the previous studies [12].The RNAi sequences targeted to MDR1 were forward, 5′-UCAUCGCAUUCC UUGCAAAdTdT-3′, and reverse, 5′-UUUGCAAGGAAUGCGAUGAdTdT-3′. These oligonucleotides were annealed after phosphorylation of their 5′ terminate, and then subcloned into pSUPER.neo+GFP to generate pSUPER-ERαi.

**Figure 8:**
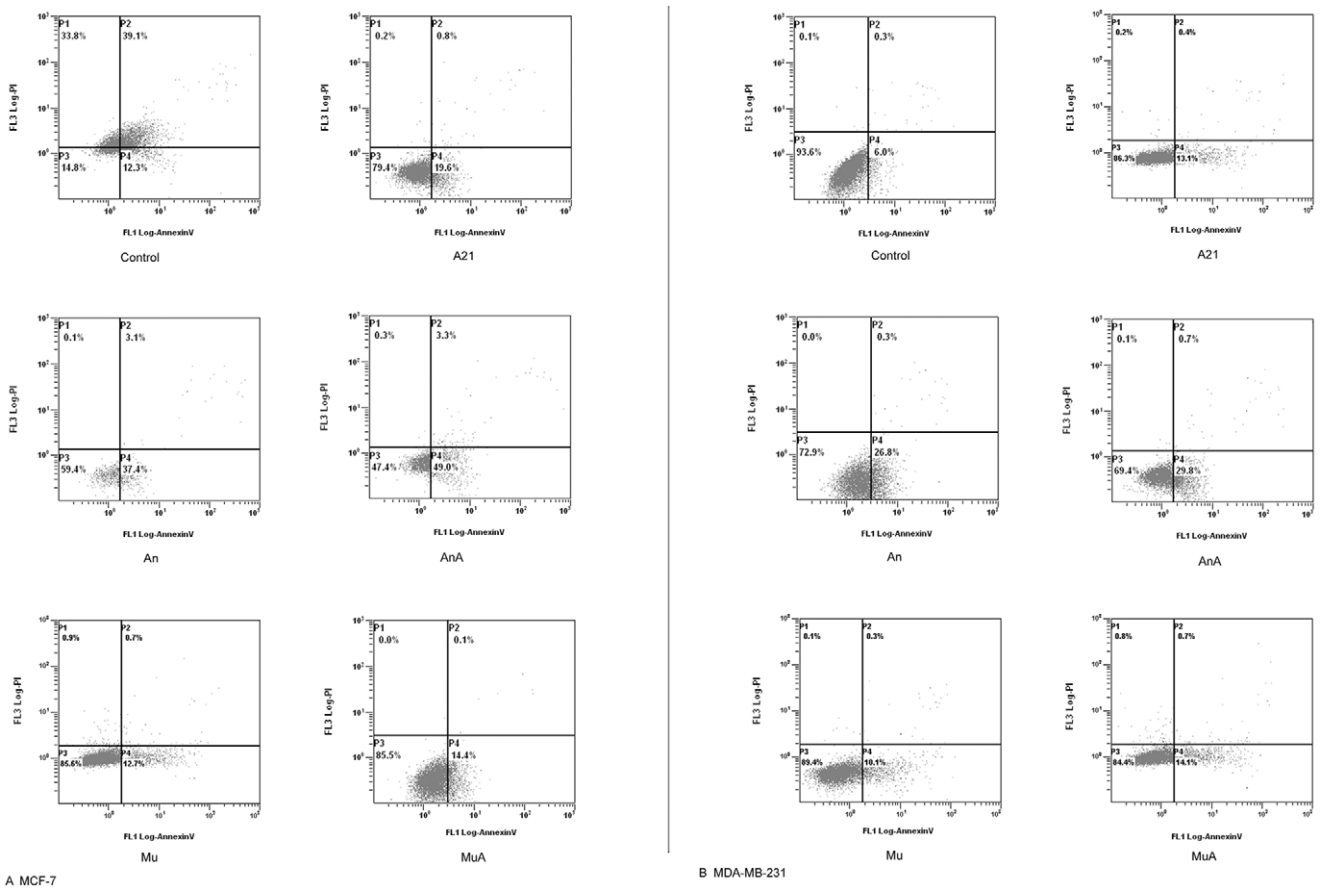
Effects of DOX on cellular apoptosis in MCF-7 and MDA-MB-231 cells. The apoptosis rate was detected by flow cytometry using the Annexin V-fluorescein isothiocyanate/propidium iodide (PI) kit, and the Annexin V-positive, PI-negative cells were scored as early apoptotic. The apoptosis rates estimated in the present study only included the early apoptotic cells which were marked as LR. A shows the alternation of apoptosis rate in MCF-7 cells and B displays that in MDA-MB-231 cells. A21, cells treated with 0.2 µmol/L DOX for 24 h; An, cells transfected with Sp1 ODNs for 24 h; AnA, cells treated with 0.2 µmol/L DOX for 24 h after their transfection with Sp1 ODNs; Mu, cells transfected with mock Sp1 ODNs for 24 h; MuA, cells treated with 0.2 µmol/L DOX for 24 h after they were transfected with mock Sp1 ODNs.

**Figure 9:**
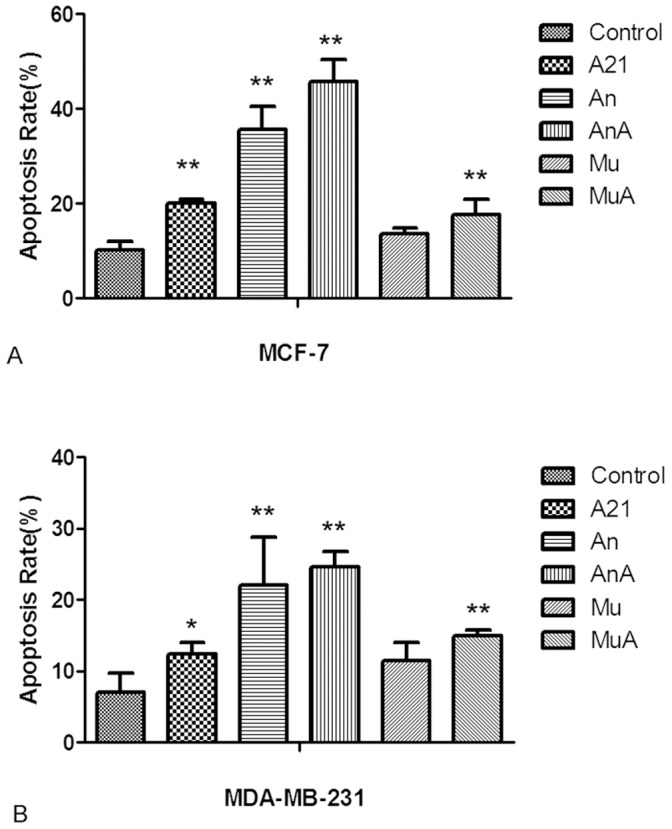
Changes on cellular apoptosis rate in MCF-7 and MDA-MB-231 cells. The apoptosis rate was detected by flow cytometry using the Annexin V-fluorescein isothiocyanate/propidium iodide (PI) kit, and the Annexin V-positive, PI-negative cells were scored as early apoptotic. The apoptosis rates estimated in the present study only included the early apoptotic cells which were marked as LR. A shows the alternation of apoptosis rate in MCF-7 cells and B displays that in MDA-MB-231 cells. A21, cells treated with 0.2 µmol/L DOX for 24 h; An, cells transfected with Sp1 ODNs for 24 h; AnA, cells treated with 0.2 µmol/L DOX for 24 h after their transfection with Sp1 ODNs; Mu, cells transfected with mock Sp1 ODNs for 24 h; MuA, cells treated with 0.2 µmol/L DOX for 24 h after they were transfected with mock Sp1 ODNs. **p<0.01 compared with control cells. *p<0.05 compared with control cells.

Before transfection, cells were seeded in 6-well plates at the density of 1×10^6^ cells per well and incubated at 37°C in an atmosphere with 5% CO_2_ for 12 h. For each well, 10 µl (2 mg/ml) of lipofectamine (Invitrogen, Carlsbad, USA) or 5 µl (1 mg/ml) of vector was diluted into 250 µl of 1640 culture medium without serum. After incubated for 10 min at room temperature, the diluted vector and lipofectamine were mixed together and incubated for 20 min. Then the mixture was adding to the cells washed three times by medium without serum. Six hours later, the medium was replaced with 1 ml of complete 1640 culture medium (The final concentration of plasmid was 5 µg/ml).As control, 10 µl of lipofectamine and 5 µl (1 mg/ml) of pSUPER.neo+GFP were also transfected.

**Table 2:** The correlation between GCS and the histopathological variables in 84 cases of invasive breast cancer.

		GCS (+) (28, 33.3%)	GCS(−) (56, 66.7%)	*p* value
Age	<35	1	5	0.658
	≥35	27	51	
Tumor stage	T1−2	25	52	0.681
	T3−4	3	4	
Nodal stage	N0	12	32	0.217
	N1−x	16	24	
Histologic rade	Grade I	2	6	0.658
	GradeII-III	26	50	
ERα	positive	24	31	0.037*
	negative	6	23	
Treatment	yes	16	17	0.018*
	No	12	39	
ERα(+)				
Treatment	yes	12	8	0.022*
	No	10	25	
ERα(−)				
Treatment	yes	4	9	0.364
	No	2	12	

* p<0.05.

ERα -estrogen receptor α.

### Cytotoxicity Assay

Assays were performed as described previously [13]. Briefly, cells were seeded in 96-well plates (1,000 cells/well) in 0.1 ml RPMI 1640 medium containing 10% FBS and cultured at 37°C for 24 h before the addition of adriamycin or mitoxantrone, both of which were obtained from Sigma, USA. Drugs were added in FBS-free medium (0.1 ml). After they were cultured for 72 h, cells were stained with 150 µl sterile MTT dye (5 mg/ml, Sigma-Aldrich, USA) for 4 h at 37°C; subsequently, the culture medium was removed and 150 µl of dimethyl sulfoxide (DMSO, Sigma-Aldrich, USA) was added and thoroughly mixed for 10 min. Absorbance at 490 nm was recorded using an automatic multiwell spectrophotometer (Bio-Rad-Coda, Richmond, CA). The MDR reversal effect was evaluated as the alteration of IC_50_.

### Apoptosis Analysis

Apoptotic rates were assessed with flow cytometry using the Annexin V-fluorescein isothiocyanate/propidium iodide (PI) kit (Bipec Biopharma Corp, USA). Samples were washed with ice-cold PBS twice and resuspended in binding buffer at a density of 1×10^6^ cells/ml. The cells were stained with Annexin V-FITC and gently vortexed. After 15 min incubation at 4–8°C in the dark, PI was added to the cells for another 5 min incubation at 4–8°C in the dark. The results were analyzed by flow cytometry (FACScan, BD Biosciences, USA).

### Statistical Analysis

The Chi-square test or the Fisher’s exact test was used to analyze the relationship between the expression of GCS and chemotherapy. Cellular data were presented as the mean ± standard deviation, and one-way ANOVA and Dunnett’s T3 was used to determine the statistical significance. P-values <0.05 were considered statistically significant. All calculations were performed using the SPSS16.0 for windows statistical software package (SPSS, Chicago, IL, USA).

## Results

### Expression of GCS Protein in the Breast Tissue Samples

The expression of GCS protein was detected in all samples by immunohistochemical staining (Figure 1). In the normal ductal epitheliums, the staining often weak and was considered negative. Overall, 33.3% of all the invasive carcinoma samples were positive for GCS (28/84); in tumors resected from patients who were treated with the CAF protocol, the expression of GCS was significantly increased (*p*  = 0.018). Further analysis showed that, in the ER-positive tumor samples, the CAF protocol increased the positive rate of GCS protein from 28.57% (10/35) to 60% (12/20) (*p*  = 0.022); however, in the ER-negative samples, CAF protocol did not induce the expression of GCS.

### Upregulation of GCS mRNA in MCF-7 Cells, but not in MDA-MB-231 Cells

qPCR was adopted to detect the expression of GCS mRNA, which was approximately 10.2-fold higher in MCF-7 cells than in MDA-MB-231 cells. As shown in Fig. 2A, DOX at 0.1 µM, 0.2 µM, and 0.7 µM for 24 h increased GCS mRNA expression in MCF-7 cells by 6.51-fold, 56.7-fold and 11.4-fold, respectively, compared with untreated MCF-7 cells. In addition, the expression of GCS after DOX treatment increased in a time-dependent manner. MCF-7 cells treated with 0.2 µmol/L DOX for 24 h had an approximate increase in GCS mRNA of 56.7-fold relative to control levels, while treatment with the same concentration of DOX for 48 h upregulated GCS mRNA levels by approximately 370-fold compared to controls.

In MDA-MB-231 cells, the low concentration of DOX (0.1 µM) decreased the level of GCS mRNA by 35.47% (p<0.01) and 0.7 µM DOX increased GCS mRNA levels by only approximately 1.34-fold relative to the controls (p<0.01) (Fig. 3A). Compared with MCF-7 cells, the effect of DOX on the expression of GCS mRNA in MDA-MB-231 cells was much smaller, and was not time-dependent.

### Alteration of GCS Protein in the Treated Cells

GCS protein expression was analyzed by Western blotting, as shown in Fig. 2B and Fig. 3B. After MCF-7 cells were treated with DOX, there was a significant increase in GCS protein expression, especially after treatment with 0.2 µM DOX for 48 h (Fig. 2B). GCS protein levels did not significantly change in MDA-MB-231 cells after DOX treatment (Fig. 3B).

### Changes in GCS Expression after Sp1 was Blocked

After cells were transfected with the Sp1 decoy ODNs, GCS mRNA levels were significantly decreased by approximately 99.97% in MCF-7 cells (Figure 4A) and 99.92% in MDA-MB-231 cells (Figure 5A) compared with the controls. Then, DOX was added to both cell lines for 24 hours. Real-time PCR and Western blotting showed that transfection with Sp1 decoy ODNs significantly inhibited the DOX-induced elevation of GCS mRNA (Figure 4) and protein levels (Figure 5) in both MCF-7 and MDA-MB-231 cells.

### Alteration of GCS mRNA and Protein in the ERα Interference MCF-7 Cells

After cells were transfected with the ERα interference plasmid pSUPER-ERαi, GCS mRNA levels were significantly decreased by approximately 84.75% in MCF-7 cells (Figure 6A) compared with the controls. Then, DOX was added to both cell lines for 24 hours. Real-time PCR and Western blotting showed that after transfected with pSUPER-ERαi, the DOX-induced elevation of both GCS mRNA (Figure 6A) and protein levels (Figure 6B) was significantly inhibited in MCF-7 cell lines.

### Evaluation of Chemosensitivity in Treated Cells

After treatment, we assessed the influence of DOX on the cellular response to anti-neoplastic drugs. The results showed that the IC_50_ of MCF-7 cells increased from 0.3155±0.0179 µmol/L to 2.164±0.0899 µmol/L after they were treated with 0.2 µM DOX for 24 hours (*p*<0.01). After they were transfected with Sp1 decoy ODNs, the IC_50_ for DOX decreased to 0.01113±0.00007 µmol/L, but did not change when cells were treated with DOX for 24 h (*p*>0.05) (Fig. 7A, Fig. 7C).

In MDA-MB-231 cells, the IC_50_ for DOX was 0.1241±0.0179 µmol/L, which increased to 0.1366±0.0116 µmol/L after they were treated with 0.2 µM DOX for 24 hours (*p*>0.05) (Fig. 7). After transfection with Sp1 decoy ODNs, the IC_50_ for DOX was reduced to 0.0114±0.0059 µmol/L and DOX could not induce the upregulation of cellular drug resistance (Fig. 7B, 7D). These combined results suggested that the Sp1/GC-rich element may play an important role in the regulation of GCS expression in the presence of DOX in MCF-7 cells, but not in MDA-MB-231 cells.

### Alteration of Apoptosis Rate in the Treated Cells

The apoptosis rate was detected by flow cytometry using the Annexin V-fluorescein isothiocyanate/propidium iodide (PI) kit. Annexin V-positive, PI-negative cells were scored as early apoptotic cells. The apoptosis rates estimated in the present study only included early apoptotic cells, which were marked as LR in Figures 8 and Figure 9. After MCF-7 cells were treated with 0.2 µM DOX for 24 hours, the rate of apoptosis increased from 10.17±1.92% to 20.0±0.87%. After transfection with Sp1 decoy ODNs, the apoptosis rate increased to 35.6±4.86%, and was unaffected by DOX (Figure 8A and Figure 9A).

In MDA-MB-231 cells, DOX increased the apoptosis rate from 7.03±2.61% to 12.4±1.67%. After transfection with Sp1 decoy ODNs, the apoptosis rate increased and the rise of apoptosis rate by DOX disappeared (Figure 8B and Figure 9B).

## Discussion

Sphingolipids, which include ceramides and sphingosine, were first isolated and characterized in the late 1800s. However, they have been long regarded as structural and insert components of cell membranes. In recent years, many studies have shown that they are still associated with a myriad of cell processes, including proliferation, cell survival and death. Ceramide, an important member of sphingolipid metabolism, has been proven to be a second messenger in the process of apoptosis [13], [14]. Cellular stress is known to increase intracellular ceramide levels. Therefore, it is easy to understand that increased ceramide levels are observed in response to many anti-cancer drugs, including doxorubicin, vincristine, paclitaxel, etoposide, PSC 833 and fenretinide.

The regulation of ceramide levels involves many enzymes, such as ceramide synthase and sphingomyelinase, which are responsible for the generation of ceramide, sphingomyelin synthase and ceramidase [8]. GCS is one enzyme that provides a major route for ceramide clearance. As an enzyme that catalyzes the first step in glycosphingolipid synthesis, GCS transfers UDP–glucose to ceramide to form glucosylceramide (GC). Increased intracellular ceramide can induce the upregulation of GCS [15], whereas the overexpression of GCS is association with decreased rates of apoptosis in many cancer types [16].

Recently, accumulating evidence has pointed towards an important role for GCS in MDR. Many drug-resistant cell lines have been found to overexpress GCS, including breast cancer cell lines, while the inhibition of GCS by antisense oligonucleotides or by specific inhibitors could restore chemosensitivity in many cancer cell lines [5]–[7]. The breast cancer resistance cell line, MCF-7/AdrR, is derived from the human ovarian carcinoma cell line, OVCAR-8, and has been re-designated NCI/ADR-RES [17]. As this cell line has been an important and widely used research tool over the last two decades, many data regarding MDR in breast cancer now have to be re-evaluated. Therefore, the function of GCS in breast cancer remains enigmatic. Our research in 2009 showed that the suppression of GCS reversed MDR in the breast cancer cell line MCF-7/ADM [10].

A GC-rich/Sp1 promoter binding region in important in the regulation of GCS expression; furthermore, doxorubicin can induce the activation of Sp1 and the upregulation of GCS and apoptosis in the leukemia drug-resistant cell line HL-60/ADR in addition to an ovarian cancer cell line [8]. Numerous data have shown that preoperative chemotherapy in breast cancer may induce the expression of many MDR-related proteins, including p-glycoprotein (p-gp), and multidrug resistance-related protein (MRP), amongst others [18]. However, there is little evidence regarding whether chemotherapeutic agents can modulate the expression of GCS in vivo. Although our study only used a relatively small number of samples, our research showed the novel result that GCS expression was significantly increased after patients were treated with the CAF protocol (*p*<0.05) (Figure 1 and Table 2).

In order to confirm whether doxorubicin induces the expression of GCS, different concentrations of doxorubicin were added to sensitive breast cancer cell lines, MCF-7 and MDA-MB-231. The results demonstrated that doxorubicin upregulated the expression of GCS at both the mRNA and protein levels in the ERα-positive cell line, MCF-7 (Figure 2), while there was no change in GCS expression in the ERα-negative cell line, MDA-MB-231 (Figure 3). We then investigated the mechanism by which doxorubicin influences GCS expression in MCF-7 cells. After blocking the Sp1 site of the GCS promoter, we found that DOX could not increase GCS mRNA and protein expression levels (Figure 4). A cellular resistance experiment (MTT assay) displayed that the drug resistance of DOX-treated MCF-7 cells significantly increased compared with untreated MCF-7 cells, and the blockade of the Sp1 site inhibited this phenomenon (Figure 7A and Figure7B). This change was consistent with the mRNA and protein results.

In 2009, Ruckhäberle et al. analyzed microarray data regarding GCS mRNA expression in 1,681 breast tumors and found that GCS expression was associated with positive estrogen receptor (ER) status, lower histological grading, low Ki67 levels and ErbB2 negativity *(p*<0.001 for all) [11]. This study revealed the expression profile of GCS in breast cancer at the mRNA level. In 2011, Liu et al. detected the levels of GCS expression in normal tissue and cancer tissue samples. Their results showed that breast and other hormone-dependent organs (testis, cervix, ovary and prostate) displayed the lowest levels of GCS mRNA, whereas the liver, kidney, bladder and stomach displayed the highest expressed levels of GCS. In breast cancer specimens, GCS overexpression is highly associated with ERα- and HER2-positivity in breast cancers that have metastasized [19]. In our study, we found that DOX induced the expression of GCS in ERα-positive MCF-7 cells significantly, but the effection on ERα-negative MDA-MB-231 cells was much smaller. This finding suggests that Erα may be related to the expression of GCS. Then we inhibited the ERα of MCF-7 cells via RNA interference, and the results displayed that the DOX-induced upregulation of GCS mRNA and protein were also been inhibited (Figure 6).

In conclusion, our data demonstrated the novel findings that DOX could modulate the expression of GCS through the Sp1 site of the GCS promoter in ERα-positive breast cancer cells.
